# Development and Validation of a Microtiter Plate-Based Assay for Determination of Bacteriophage Host Range and Virulence

**DOI:** 10.3390/v10040189

**Published:** 2018-04-12

**Authors:** Yicheng Xie, Laith Wahab, Jason J. Gill

**Affiliations:** 1 Department of Animal Science, Texas A&M University, College Station, TX 77843, USA; seasonxie@tamu.edu (Y.X.); laithwahab@tamu.edu (L.W.); 2 Veterinary Physiology and Pharmacology, Texas A&M University, College Station, TX 77843, USA; 3 Center for Phage Technology, Texas A&M University, College Station, TX 77843, USA

**Keywords:** bacteriophage, host range, *Salmonella*, method development

## Abstract

Bacteriophages, which are the natural predators of bacteria, have re-emerged as an attractive alternative to combat antibiotic resistant bacteria. Phages are highly specific at the species and strain level and measurement of the phage host range plays an important role in utilizing the phage as antimicrobials. The most common method for phage host range determination has been to spot phage lysates on soft agar overlays and observe plaque formation. In this study, a liquid culture-based assay was developed in a 96-well microtiter plate format to measure the phage host range and virulence for a collection of 15 *Salmonella* phages against a panel of 20 *Salmonella* strains representing 11 serovars. This method was compared to a traditional spot method. The majority of the host range results from two methods were in agreement including in cases where a bacterial strain was insensitive to the phage. Each method produced a false-negative result in 19/300 (6%) of the measured phage-host combinations when compared to the other method. The spot method tended to indicate greater phage sensitivity than the microtiter assay even though direct comparisons of the response magnitude between the two methods is difficult since they operate on different mechanisms. The microtiter plate assay was able to provide data on both the phage host range and virulence in greater resolution in a high-throughput format.

## 1. Introduction

The growing threat of antibiotic resistance has led to increased calls for new antimicrobials to control bacterial pathogens and treat infectious diseases [1,2,3]. Bacteriophages are the natural predators of bacteria and were used to treat bacterial infections in the pre-antibiotic era [4,5]. Phages have re-emerged as an attractive alternative to combat antibiotic resistant bacteria in recent decades [6]. The abundance of phages in natural environments makes the discovery and isolation process rather simple [7,8], but screening and selecting the right phages is crucial for achieving successful therapeutic outcomes. One advantage of phages as therapeutics is their host specificity [9,10] and the ability of most phages to infect only a relatively narrow range of closely related bacterial strains limits its impact on normal bacterial flora [9,11]. The drawback to host specificity is the limitation on treatment outcomes prior to identifying bacterial susceptibility of particular phages [9].

The ability of a phage to infect and lyse the target bacterial strain is generally agreed to be a basic requirement for successful phage therapy [12,13,14]. Phage virulence, which is defined as the ability of a phage to control the growth of its host in culture, may also be an indicator of phage utility [15,16,17]. The phage host range is affected by a number of factors [18,19]. The phage must be able to adsorb to the cell surface in order to initiate infection and the absence or masking of a compatible cell surface receptor will prevent this initial interaction. With successful phage adsorption, the entry of phage DNA to a bacterial cell could be blocked by superinfection exclusion systems or the absence of required accessory proteins. Restriction-modification and CRISPR systems block infection by degrading phage DNA shortly after it arrives in the cell cytoplasm and abortive infection systems that are triggered on phage infection result in host cell death before new phages can be produced. All of these factors together limit the host ranges of naturally recurring phages and can affect the results of experiments designed to determine the phage host range. Given the increased interest in phage therapy, there is a need for in vitro assays that can be used to help determine the suitability of phages for in vivo application [17,20].

The definitions and outcomes of the phage host range vary between testing methodologies [10,21]. To claim a host is “sensitive” to a phage may mean that the phage is able to infect, produce progeny, and lyse its host or simply that the host cell dies following phage infection. The spot test is often used to determine the host range by measuring bacterial killing by applying high-titer phage lysates to agar lawns inoculated with host bacteria [22]. Applying phage only at high titer can fail to distinguish between the ability of phage to replicate within or simply kill the test strain since a similar result could be produced through phage infection and lysis, abortive infection, or lysis from without [10]. Spotting single dilutions of lower phage concentrations has also been a common host range method [23,24]. In this approach, the phage is diluted to a routine test dilution (RTD), which is typically defined as the lowest phage dilution that still forms a zone of lysis on its propagation host. This method is more sensitive than spotting a high-titer phage lysate since a level of productive phage infection is required to produce a signal. Another commonly used method for testing the host range is measuring phage efficiency of plating (EOP), which counts the number of plaques formed by a phage on a test strain relative to its titer observed on its original host [10,22]. Plaque formation is a better indicator of productive phage infection since it is the result of multiple rounds of infection, lysis, and release of progeny. Mirzaei and Nilsson [25] compared the results from spotting high titer lysates and measuring EOP in phages infecting *E. coli* and *Salmonella*. The researchers determined that the high-titer spotting method often overestimated the phage host range and virulence. While the production of clearing zones in bacterial lawns following application of high-titer phage lysate may overestimate phage sensitivity, the inability of a phage to form visible plaques in a bacterial lawn does not necessarily mean a lack of productive infection. Plaque formation is a dynamic process and differences in phage latent period, burst size, diffusion rate, and growth of the host can all affect plaque size and visibility [10,26]. Observation of phage infection in a liquid culture could serve as an alternative method for measuring the phage host range and virulence for phages that are incapable of making observable plaques [10]. Henry, Lavigne, and Debarbieux [15] analyzed phage virulence in *Pseudomonas aeruginosa* broth culture and were able to differentiate between phages based on differences in the culture optical density over time. In this case, all phages studied were already known to infect the host strain by EOP-type assays. However, performing liquid culture assays in traditional culture flasks is time and labor intensive and limits the throughput of the method.

Minimum inhibitory concentration (MIC) assays are typically used to simultaneously test multiple chemical antimicrobials against target organisms in a high throughput format such as the standard 96-well microtiter plate [27]. In this approach, a culture of the test organism is inoculated at a low density (~10^5^–10^6^ CFU/mL) into wells containing graded concentrations of antimicrobial compounds in broth medium. The presence or absence of bacterial growth is scored following a fixed period of incubation. The culture containing the lowest concentration of antimicrobial that inhibits bacterial growth is termed the MIC of the compound for that organism. This approach has been expanded to estimate the performance of other biocidal compounds in food safety applications [28,29]. Endpoint MIC-like assays have been adopted to measure the host range and virulence of phages infecting enterohemorrhagic *E. coli* [30] and phages of *S. aureus*, *E. coli*, and *Salmonella* [31]. However, such endpoint assays can yield false-negative results if phage resistance acquired during the course of the assay results in culture turbidity at the time of end-point determination [10]. Phages that are capable of infecting the host but do not replicate well in liquid culture could fail to clear the culture. This also leads to a false negative at end point determination [10]. The Bioscreen-C growth analysis system has been used to obtain real-time measurement of culture optical density in the presence of phages to determine phage virulence [32] and host range [33,34,35]. The Bioscreen-C system can provide high-resolution monitoring of culture optical densities over time and these assays have been evaluated qualitatively or by measuring the inflection of the growth curve at set time points. The measurement of bacterial respiration in the presence of phage in liquid culture has also been used to simultaneously determine phage host range and virulence in a high-throughput, 96-well format [36]. In this system, the production of a colorimetric signal by reducing a tetrazolium dye is measured instead of the culture’s optical density [36].

In the current study, a liquid culture-based host range method is developed, which continuously monitors bacterial growth in the presence of phage in a standard 96-well format and this method is compared to the results from conventional agar overlay spot assays. The intent of this methodology is to determine the phage host range, virulence, and bacterial resistance development in a single high-throughput format by using the features of an automated plate reader to monitor the culture optical density over time in an incubating, aerated environment. Growth responses are quantified by integrating the growth curve over the entire experiment, which allows them to be directly compared. This microtiter plate host range assay is expected to serve as an alternative host range method and can potentially be a more sensitive predictor of virulence of phages by providing more information on bacterial inhibition with high resolution between bacterial strains.

## 2. Materials and Methods

### 2.1. Bacterial Strains and Culture Conditions

A panel of 20 *Salmonella* strains from various sources representing 11 serovars were used in this study, which is shown in Table 1. All bacteria and phages were cultured in tryptic soy broth (TSB) (Becton-Dickinson, Franklin Lakes, NJ, USA) or tryptic soy agar (TSB plus 1.5% *w/v* Bacto agar (Becton-Dickinson)) aerobically at 37 °C.

### 2.2. Bacteriophage Strains and Culture Conditions

Phage FelixO1 was obtained from the *Salmonella* Genetic Stock Center (University of Calgary, Calgary, AB, Canada) and propagated on the *S.* Typhimurium strain LT2. Twelve of the phages used in this study were isolated in 2014 from a set of 72 phage-enriched beef feedlot environmental samples described previously [37]. The 72 enrichments were pooled by pen, feedlot, and enrichment strains to produce 18 composite samples and enriched again against the same two sets of mixed bacterial hosts, which was described previously [37]. Two additional phages known as Sw2 and Melville were isolated in 2016 from a municipal wastewater influent sample enriched for phage using the same mixed-host panel as above. Each enrichment was serially diluted and plated to soft agar lawns inoculated with each individual enrichment host. Aditionally, individual plaques were picked and purified by subculturing three times. Plaques picked from the third subculture of each bacteriophage were used to produce high-titer phage stocks by using the confluent plate lysate method [40]. Soft agar overlays were prepared as described below. Phage stocks were stored and diluted in phage buffer (100 mM NaCl, 25 mM Tris-HCl pH 7.4, 8 mM MgSO_4_, 0.01% *w/v* gelatin) at 4 °C. Phages used in this study and their propagation hosts are summarized in Table 2.

### 2.3. Phage Host Range Agar Overlay Spot Assay

Fresh (~18 h) overnight cultures of bacterial strains were prepared in TSB, subcultured 1:100 in fresh TSB, and grown to OD_550_ ~0.5. Agar overlays were prepared by inoculating 4 mL of molten top agar (10 g L^−1^ Bacto tryptone (Becton-Dickinson), 10 g L^−1^ NaCl, 5 g L^−1^ Bacto agar) with 100 µL of OD_550_ ~0.5 host culture and then poured over TSA plates. Lysates of each phage were ten-fold serial diluted and 10 µL of each dilution was spotted onto the overlay inoculated with the original host of each phage, dried, and incubated at 37 °C overnight [22]. In this study, the routine test dilution (RTD) [23] was defined as the first dilution at which the phage produced countable plaques on a lawn of their propagation host. Each phage lysate was adjusted with phage buffer to achieve the RTD and 100× RTD, spotted on the overlays of the panel of 20 *Salmonella* strains, and incubated at 37 °C overnight. Spot assay results of each phage-bacterium combination were scored using the following parameters. Production of >50% of the plaques formed on the propagation host at RTD = 4; production of <50% of the number of plaques formed on the propagation host at RTD = 3; production of a confluent zone of lysis but no individual plaques at 100× RTD = 2; production of individual plaques at 100× RTD = 1; and no plaque formation at either dilution was scored as 0.

### 2.4. Methodology Development for the Microtiter Plate Host Range Assay

A subset of four bacterial strains (*S.* Anatum strain FC1033C3, *S.* Newport strain USDA2, *S.*Typhimurium strain USDA1, and *S.* Enteritidis strain SGSC 2475) and four phages (Sasha, Season12, Munch, and Sw2) were selected to develop the parameters for the microtiter plate liquid-culture host range assay. Different initial bacterial inoculum levels were tested in combination with phages at starting concentrations of 10^6^ to 10^8^ PFU/mL. The low inoculum condition (~10^5^ CFU/mL) was achieved by adjusting fresh overnight cultures OD_550nm_ ~0.5 and diluting 1000-fold in TSB. For the high inoculum condition, fresh overnight cultures were adjusted with TSB to OD_550nm_ ~0.1 to achieve a concentration of ~10^8^ CFU/mL. Phage lysates were titered and adjusted to concentrations of 10^7^, 10^8^, and 10^9^ PFU/mL with phage buffer. For each assay, 180 µL of adjusted bacterial inocula in TSB were mixed with 20 µL of phage in sterile, untreated Falcon (Corning) 96-well transparent plates to achieve final phage concentrations of 10^6^ PFU/mL, 10^7^ PFU/mL, and 10^8^ PFU/mL. The plates were incubated at 37 °C with double orbital shaking in a Tecan Spark 10 M plate reader (Tecan Group Ltd., Männedorf, Switzerland) and growth was monitored by measuring OD_550nm_ at 30-min intervals for 12 h, which results in 25 total time points including the initial (time 0) measurement. Growth curves were obtained by plotting OD after baseline adjustment against time. All assays were performed with three biological replicates.

### 2.5. Analysis of Microtiter Plate Host Range Assay Data

**Figure 1:**
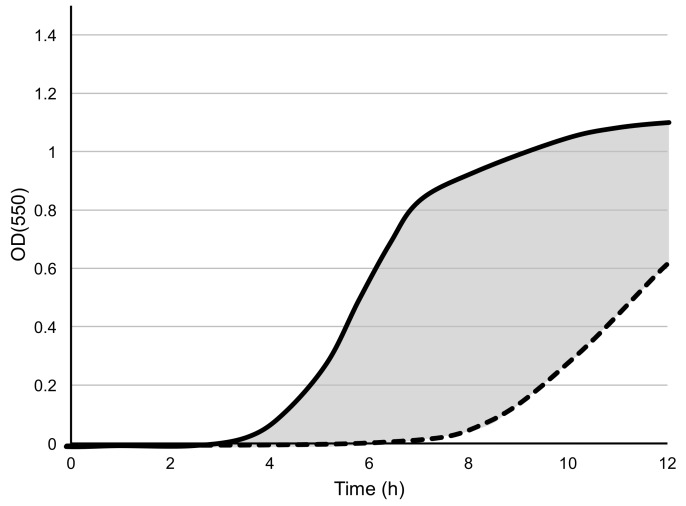
An illustration of the liquid assay score is derived by Equation (2). The equation calculates the area (grey) between the positive control (solid line) and the phage-inoculated culture (dashed line) and expresses this as a percentage of the total area under the positive control curve. No inhibition of bacterial growth (solid and dashed lines overlap) results in a score of zero and completes the absence of growth in the phage-inoculated culture (dashed line follows X-axis) would result in a score of 100.

Based on the results obtained from the above pilot experiments, the high bacterial inoculum (~10^8^ CFU/mL) condition with phages at 10^6^ and 10^8^ PFU/mL was used to assess the phage host range and virulence for the rest of the collection. Preparation of bacterial inoculum, phage, and measurement parameters were as described above in triplicate. To facilitate data analysis, the growth patterns observed in each assay were distilled into single numerical values by measuring the area under each curve for both positive control and phage treatments by using the equation below.

(1) $$Area\;under\;the\;curve=\;\overset{24}{\underset{i=1}{\sum }}\frac{OD_{i+1}+OD_{i}}{2}$$

where *OD* were measured at 550 nm at 30-min time points *i.* For example, the OD_550nm_ measured at time timepoint *i* = 3 (60 min into the experiment) is added to the OD_550nm_ measured at *i* = 4 (90 min) and divided by 2 to give the OD_550nm_ value for the center of the interval. Each of these values are combined over the entire experiment to give a total area under the growth curve. Since all time intervals are equal in this procedure, time is not explicitly required in this calculation. The areas under the curve calculated in Equation (1) are normalized as percentages of the area under the curve of the positive control by Equation (2).

(2) $$Liquid\;assay\;score=\;\frac{Area_{positive\;control}-Area_{phage\;treatment}}{Area_{positive\;control}}\times 100$$

where the *Area_positive control_* and *Area_phage treatment_* are the areas under each curve obtained from Equation (1). The *liquid assay score* is equal to the area between the positive control curve and phage treatment curve, divided by the total area below positive control curve, and multiplied by 100. An illustration of the derivation of the assay score is shown in Figure 1. In this way, the assay scores represent how well phages are able to suppress bacterial growth during the 12-h experiment. The average values calculated by Equation (2) across triplicate biological replicates (*n* = 3) were used as the assay scores for all phage-host combinations.

Comparisons between the microtiter plate assay and spotting assay results for each phage-host combination were carried out by normalizing the results of both methods to the result obtained for the phage propagation host and calculating the difference using the equation below.

(3) $$\begin{array}{cc} Difference\;score & =\frac{Spot\;assay\;score}{Spot\;assay\;score\;obtained\;on\;its\;host}\times 100\\ & -\frac{Liquid\;assay\;score}{Liquid\;assay\;score\;obtained\;on\;its\;host}\times 100 \end{array}$$

The calculated values of difference demonstrate the agreement or disagreement between two host range methods with greater positive values, which indicates higher phage sensitivity measured by the spot assay, and greater negative values indicate higher sensitivity measured by the microtiter assay. In each case, one spot assay score was compared to two microtiter assay scores at different phage concentrations to determine if one condition yielded higher agreement with the spotting assay.

### 2.6. Statistical Analysis

Statistical analyses were performed using JMP Pro v12 (SAS Inst. Inc., Cary, NC, USA). The Shapiro-Wilk Test was performed to determine the normality of distribution for microtiter assay scores by concentrations across all tested bacterial strains. Significantly differing assay scores by concentrations were separated by using the Wilcoxon/Kruskal Wallis Test (*p* < 0.05).

## 3. Results and Discussion

### 3.1. Measurement of Phage Host Range by Traditional Spot Assay

**Figure 2:**
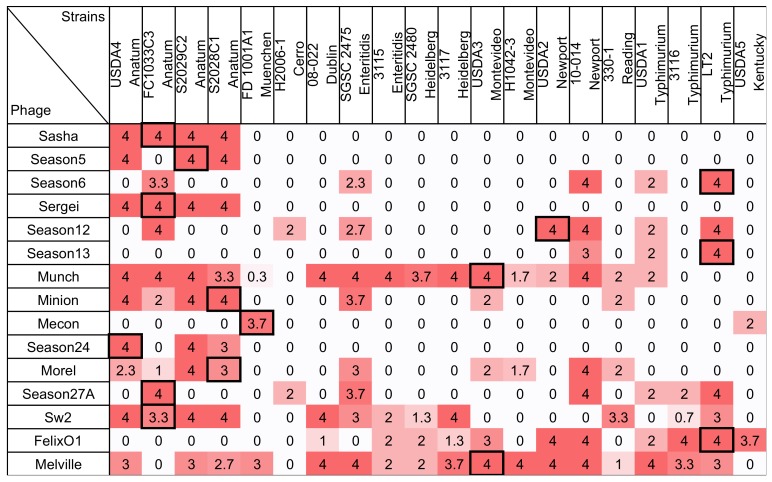
The host range of 15 *Salmonella* phages against 20 *Salmonella* strains were measured by spotting on soft agar overlays. Phages were plated at the routine test dilution (RTD and determined as the first tenfold serial dilution of phage lysate that formed countable plaques on lawns of its own host) and 100× the RTD. Phage were tested and scored on the following criteria: phage forming >50% of the number of plaques formed on its host strain at its RTD = 4; phage forming 5% to 50% of the number of plaques formed on its host strain at its RTD = 3; phage forming a zone of confluent lysis but no individual plaques at 100× RTD = 2; phage forming individual plaques at 100× RTD = 1; and no plaque or clearing formation at either dilution = 0. The scores from three replicate experiments were averaged. To aid the reader, cells are shaded with stronger color intensity indicating a greater score. Boxed cells indicate the initial isolation and propagation host of the phage. For clarity, all values are displayed to one significant figure unless a non-zero value is present at the first decimal place.

The host ranges of 15 *Salmonella* phages against a panel of 20 *Salmonella* strains, which is measured by a traditional spot assay, are shown in Figure 2. In this method, phages adjusted to a consistent routine test dilution (RTD) were spotted to agar overlays inoculated with each bacterial test strain and observed for the formation of plaques. A scoring system was used to summarize plaque formation by spotting the phage lysates at the RTD and at 100× RTD. A score of 4 corresponds to phage efficiency of plating (EOP, the number of plaques observed on the test strain divided by the number of plaques observed on the phage host) of ~0.5 to 1, a score of 3 represents an EOP of ~0.05–0.5, a score of 2 represents an EOP of ~0.01–0.05, and a score of 1 represents an EOP of ~0.0002–0.01. Scores of 0 represent phage EOP of less than ~0.0002. The *Salmonella* strain panel used in this study was diverse and represents 11 serotypes that are commonly associated with human disease or animal carriage [39]. The host ranges of the tested phages were highly variable, which infects from 10% to 85% of the tested *Salmonella* strains. Phage Melville was found to have the broadest host range since it is capable of infecting 85% (17/20) of strains tested and able to efficiently infect (defined here as a score >3, which corresponds to an EOP of ~0.05–0.5) 13 strains. Phage Mecon was found to have the narrowest host range by using this method, which capable of efficiently infecting only its own host and lysing one other strain.

Phage Felix O1, a well-studied broad host range phage, was previously shown to lyse 85.3% (191/224) of *Salmonella* strains and 5.9% of *Escherichia coli* strains when tested by a spot assay at a concentration 6 × 10^10^ PFU/mL [41]. In the present study, this phage was able to infect only 55% (11/20) *Salmonella* strains tested when applied at concentrations of ~10^5^ PFU/mL or less. This difference illustrates the importance of assay parameters in estimating the host ranges of phages since application of high phage titers can tend to overestimate bacterial sensitivity to phage [10,22]. Consistent with this observation, Welkos et al. (1974) further showed that spot testing Felix O1 at 10^12^ PFU/mL resulted in an increase of the apparent sensitivity of *Salmonella* strains to 98.5%.

Defined as the highest dilution for the phage to produce countable plaques on its propagation host, the phage titer of the RTD in this study is slightly lower than the method described by Wilson and Artkinson [23] in which RTD was defined the lowest serial dilution for the phage to produce a confluent zone of lysis. By using both RTD and 100× RTD, this method was designed to test the ability of phages to replicate on a host strain without the false positive results due to abortive infection and lysis. Without that, it can be seen when high phage titers are applied [10] while also providing a simplified score for of EOP that is restricted to a ~2 log_10_ range [10,22]. The scoring system used in this study also helps in methodology comparison by providing a numerically consistent, semi-quantitative result.

### 3.2. Determination of Microtiter Assay Parameters

**Figure 3:**
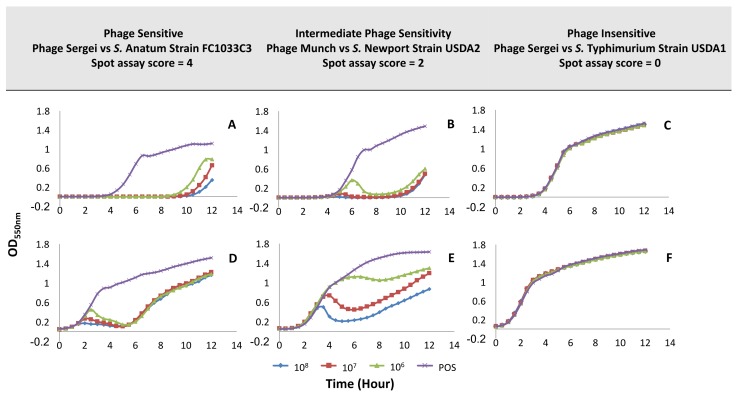
Sample growth curves illustrating different phage sensitivity phenotypes in the low- and high-inoculum experimental setups. The X-axis indicates the OD_550nm_ and the Y-axis represents the time in hours. Panels (**A**–**C**): experiments run with the low bacterial inoculum condition (~10^5^ CFU/mL); panels (**D**–**F**): experiments run with the high bacterial inoculum condition (~10^8^ CFU/mL). Both conditions were challenged with phage at 10^8^ PFU/mL, 10^7^ PFU/mL, and 10^6^ PFU/mL; POS: positive culture control with no phage. Phage-host pairs were selected based on their scores from the spot host range assay, which was shown in Figure 1.

Based on the results of the spot host range assay, pilot experiments that explored the parameters for a liquid culture-based host range assay were performed. Phage-host combinations representing sensitive, intermediate, and resistant phenotypes were selected to evaluate the performance of different levels of bacterial and phage inocula in a 96-well microplate format assay (see Figure 3).

Figure 3A,D, which show the growth of the *S.* Anatum strain FC1033C3 in the presence of phage Sasha at low (~10^5^ CFU/mL) and high (~10^8^ CFU/mL) bacterial inoculum levels, represent the scenario of high bacterial sensitivity to phage with a score of 4 (EOP ~ 1) in the spot host range assay (see Figure 2). The initial bacterial inoculum level had an effect on the shape of the bacterial growth curve, but in both experiments, the phage was able to produce strong control of growth in liquid culture. The level of phage inoculum had only a minor effect on the bacterial growth phenotype. In the low-inoculum condition (see Figure 3A), phages were able to suppress bacterial growth up to approximately nine hours while in the high-inoculum condition (Figure 3D), inhibition of bacterial growth was not observed until 1–2 h of the experiment with OD_550_ reaching a minimum at five hours followed by bacterial regrowth at the end of the experiment. In the low inoculum condition, the input multiplicity of infection (MOI) is relatively high (~10–10^3^ PFU:CFU). Therefore, the shape of the growth curve phage likely reflects an initial killing of most of the bacterial population followed by the regrowth of a phage-insensitive mutant population after 9–10 h. On the other hand, the input MOI in the high inoculum condition is relatively low (~0.01–1 PFU:CFU), so significant lysis of the bacterial population cannot occur until the input phages have undergone multiple replication cycles to reach high enough concentrations to infect the majority of cells in the culture. When the bacterial host is highly sensitive to the phage, the low inoculum condition appears to mainly show the time until the arisal of a phage-resistant population while giving little information on factors such as the rate of phage replication. However, by using the high inoculum method, the phage must be able to replicate fast enough to outnumber, infect, and lyse a large proportion of the bacterial population in order to observe a significant reduction in OD_550_, which provides more information on phage virulence.

A scenario of intermediate bacterial sensitivity to phage was evaluated using *S.* Newport strain USDA2 and phage Munch (spot assay score of 2, Figure 2). In the low bacterial inoculum condition (see Figure 3B), growth curves at phage concentrations of 10^7^ and 10^8^ PFU/mL were similar to those observed for the highly sensitive phage-host pair shown in Figure 3A. At the lowest phage concentration (10^6^ PFU/mL, Figure 3B), the OD_550_ rose similar the positive control peaked at six hours and then dropped until the regrowth of the culture at ~10 h. In the high bacterial inoculum condition (see Figure 3E), the bacterial growth curve was qualitatively different from the sensitive phage-host pair (see Figure 3D), which produces a dose-dependent reduction in bacterial growth that was similar to the sensitive host at high phage concentration but weaker at the lower phage concentrations. This combination represents a situation in which the phage is able to infect its host, replicate, and produce progeny, but it may not be able to accomplish this as efficiently as observed in the Sasha/FC1033C3 phage-host pair. This observation demonstrates that the high inoculum condition provides greater discriminatory power between intermediate phage sensitivity phenotypes.

Phage Sasha and *S.* Typhimurium strain USDA1 (see Figure 3C,F), which represents bacterial insensitivity to phage with a spot assay score of zero (see Figure 2), yielded no observable effects of phage on bacterial growth under any condition. This would be the result expected in the case of true phage resistance where the phage is unable to interact with the bacterium and has no effect on its growth.

Based on the observations of bacterial growth inhibition in Figure 3, the high bacterial inoculum condition (~10^8^ CFU/mL) with phage inoculation at 10^8^ PFU/mL and 10^6^ PFU/mL (corresponding to multiplicities of infection of ~1 and 0.01, respectively) were selected for conducting microtiter host range assays for the remainder of the phage collection. These parameters appeared to provide the greatest discriminatory power between high and intermediate phage sensitivity phenotypes, which was illustrated by the differences between Figure 3D and Figure 3E. The high bacterial inoculum condition demonstrated dosage effects of phage treatment with a higher observable effect on growth than was observed in the low inoculum condition. Phage concentrations of 10^8^ PFU/mL and 10^6^ PFU/mL were selected for further assays in order to simplify the method since the 10^7^ PFU/mL condition did not appear to add any additional discriminatory power.

### 3.3. Measurement of Phage Host Range and Virulence by Microtiter Plate Assay

**Figure 4:**
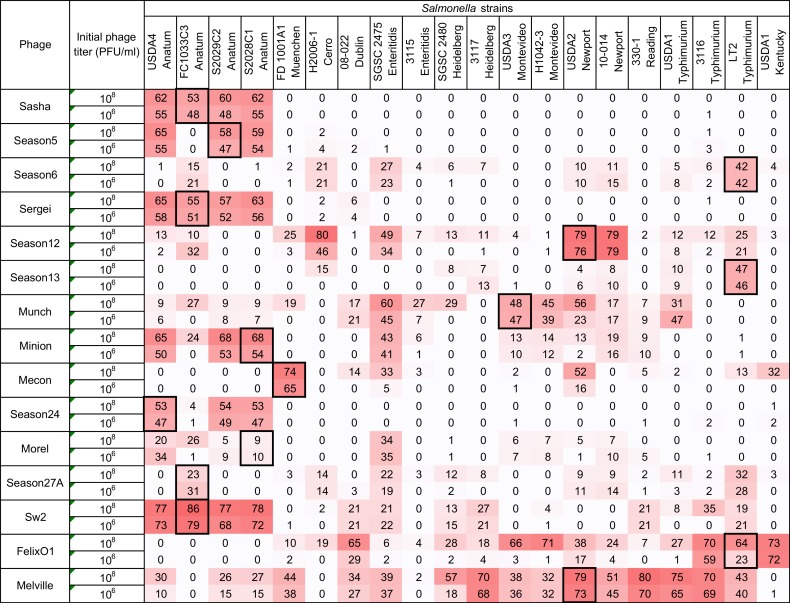
Host range of 15 *Salmonella* phages against 20 *Salmonella* strains is determined by the microtiter plate liquid assay at two initial phage concentrations of 10^6^ PFU/mL and 10^8^ PFU/mL. Values indicate average liquid assay scores calculated by Equation (2) across three replicate experiments. To aid the reader, cells are shaded with stronger color intensity indicating a greater score. Assay score represents the differences in area under the bacterial growth curve with and without phage. Larger numbers indicate a greater suppression of bacterial growth in the presence of phage. Boxed cells indicate the initial isolation and propagation host of the phage. Standard deviations of each experimental unit across this assay ranged from 0.03 to 10.89. Negative values falling within one standard deviation were adjusted to zero for the convenience and for calculating the following comparison between two host range methods.

Using the high inoculum condition and two phage concentrations as described above, the remaining 297 phage-host combinations were tested in a liquid culture-based host range assay in the 96-well microtiter plate format. To simplify the display of this data and facilitate comparisons, each growth curve was transformed (as shown in Equation (2) and Figure 1) into a single value representing the difference under the growth curves between the phage treatment and the positive control (see Figure 4). Since the largest standard deviation observed in any individual assay was 10.89, an assay score of greater than 10.9 was used as a cutoff to distinguish a legitimate signal from noise. Using this simple cutoff, 186 individual assays produced a positive signal in this system. Similar to the results found in the spot host range assay (see Figure 2), phage Melville displayed the broadest host range capable of infecting 16/20 (80%) of hosts tested (see Figure 4) with the highest score of 80 against *S.* Reading strain 330-1 at a phage inoculum of 10^8^ PFU/mL.

A phage dosage effect was observed across this assay with greater bacterial growth suppression observed in the high phage concentration (10^8^ PFU/mL) condition. As an example, phage Felix O1 was able to suppress bacterial growth in 25% (5/20) strains at a phage concentration 10^6^ CFU/mL and the observed suppression expanded to 60% (12/20) of strains when the phage concentration was increased to 10^8^ CFU/mL. The greatest difference in bacterial growth between the two tested phage concentrations was in the combination of phage Felix O1 against *S.* Montevideo strain H1042-3 in which the low phage concentration produced a score of 1 (bacterial growth almost identical to the no-phage control) and the high concentration produced a score of 71 (strong suppression of bacterial growth). Across all assays, the 10^6^ PFU/mL phage inoculum produced 80 positive results (score > 10.9) while the 10^8^ PFU/mL inoculum produced 106 positive results. The overall distribution of microtiter assay scores was statistically greater in the assays with higher phage concentration (Wilcoxon/Kruskal Wallis Test, *p* = 0.0141). This dosage effect is similar in principle to the observations of Welkos et al. [41] in the traditional spot assay where the testing phage at higher titer resulted in an increased apparent sensitivity to phage in *Salmonella*. This observation highlights the importance of the assay parameters on the observed phenotypes and the value of using multiple initial phage concentrations to ascertain phage host range and virulence in such assays.

An advantage of liquid culture assays over the traditional spot assay is that they measure the ability of a phage to control bacterial growth over time, which is a property typically referred to as phage virulence [42]. Measurement of virulence in this sense is an integrated result of a phage’s ability to infect, reproduce within, and lyse a bacterial host. Phages with high adsorption rate constants, short latent periods, and large burst sizes would be expected to produce a stronger signal in this type of experiment [43,44]. In the case of the microtiter assay, multiple comparisons between phage-host pairs can be made because of the standardization of the assay and the mathematical transformation of the resulting bacterial growth curves into single numerical values. This assay can, therefore, determine the ability of a phage to interact with a bacterial strain at a detectable level (the phage “host range”) and also measure the ability of a phage to control a bacterial population in a liquid culture (phage “virulence”).

### 3.4. Comparison Between Two Host Range Methods

**Figure 5:**
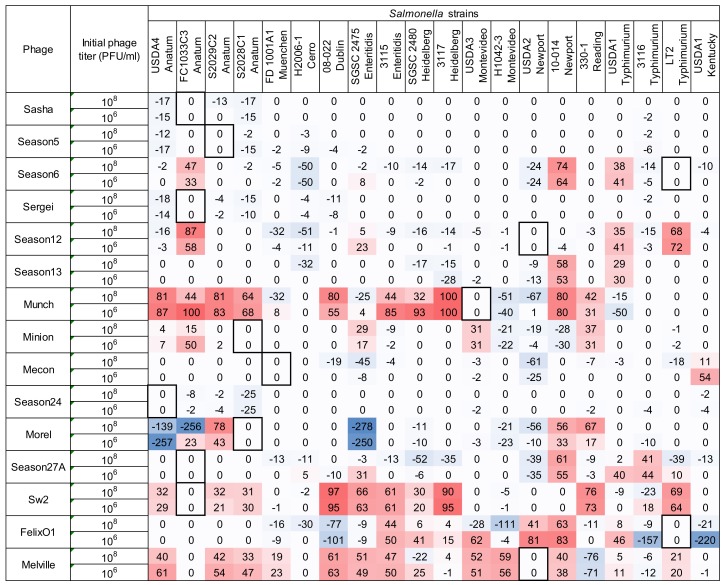
Agreement levels between two host range methods. Equation (3) was used to determine agreement levels between two host range methods. One spot assay score representing one bacteria-phage combination (see Figure 1) was compared to two liquid assay scores at two different phage concentrations (see Figure 3) to determine if one phage concentration in the liquid assay yielded a difference in agreement levels. Values closer to zero indicate greater agreement between the two methods and values closer to 100 indicate disagreement. Negative values indicate greater bacterial sensitivity to phages observed in the liquid assay and positive values indicate greater sensitivity observed in the spot assay. Color intensity indicates greater divergence from zero in either direction, with blue shades indicating negative values and red shades indicating positive values. Boxed cells indicate the initial isolation and propagation host of the phage.

The differences between the two host range methods are shown in Figure 5, which was calculated by Equation (3). Difference scores close to zero of either sign indicate high agreement between the two methods and greater deviations from zero indicate greater disagreement. Negative values (blue) indicate a greater response was observed in the microtiter plate assay while positive values (red) indicate a stronger response in the spot assay.

Among these 600 points of comparison (fifteen phages against twenty bacterial strains at two concentrations), the majority of the assay results (74%, 444/600) showed high agreement in which their difference scores ranged between an arbitrary limit of −20 to +20 while only 4.7% (28/600) showed high disagreement with difference scores of +/−80 or more. Among 300 phage-host combinations, over half (171/300, or 57%) of the total results were cases where both methods produced a result of phage insensitivity (score of 0 in the spot assay and less than 10.9 in the microtiter assay), which indicates that the two methods tend to support each other in answering the question of whether a bacterial strain is sensitive or insensitive to a given phage. In 19 phage-host combinations, the spot assay indicated phage insensitivity (score = 0) but the microtiter assay produced a detectable response (score > 10.9). At the same time, 19 other phage-host combinations showed no response (score < 10.9) in the microtiter assay but a detectable response (score > 0) in the spot assay. This supports the finding that both methods are generally equally likely to detect phage sensitivity across phage-host pairs, but any given phage-host pair could show a false-negative result (compared to the other method) approximately 6% of the time in either assay. Differences in detecting phage sensitivity between the two methods were not evenly distributed across phage-host pairs. In the spot assay, 16/19 (84%) of false-negative results (where the spot assay score was 0 but the microtiter assay score was >10.9) were confined to the results of five phage isolates. In the microtiter assay, 11/19 (58%) of false-negative results (where the microtiter assay score was less than 10.9 but the spot assay score was >0) were confined to only two phages. This indicates certain phage isolates may fail to provide a response in either method but this can only be determined empirically. For example, phage Munch was capable of producing a response on 16 of 20 tested strains in the spot assay (see Figure 2), but this was modulating bacterial growth in the microtiter assay in 11/20 strains at the 10^8^ PFU/mL inoculum and in 7/20 strains at the lower 10^6^ PFU/mL inoculum (see Figure 5). While the lack of signal does not definitively rule out the ability of a phage to interact with the test strain, it does indicate that the phage cannot efficiently infect and lyse the strain, which is a primary concern when selecting phages for potential use in therapeutic applications.

When disagreements arose between the two methods, the spot assay tended to indicate higher levels of phage sensitivity. Of the 156 difference scores greater than 20 or less than −20 (see Figure 5), 108 (69%) were positive, which shows a greater response in the spot assay than in the microplate assay. In the level of high disagreement (difference scores greater than ±80), 19 out of 28 difference scores (68%) were positive, which shows that the ability of a phage to form plaques or clear zones on agar plates does not always confer the ability to suppress bacterial growth in liquid culture. For example, phage Munch, which showed high efficiency of plating against four *S.* Anatum strains in the spot assay with scores of 3.3 to 4 (see Figure 2), largely failed to inhibit bacterial growth of those strains in the microtiter assay (see Figure 4). The disagreement between the two methods also appeared to be associated with broad host-range phages and with slightly more than half (87/156) of all disagreement scores ≥ ±20 associated with only four phages: Munch, Sw2, FelixO1, and Melville.

The ability of a phage to suppress bacterial growth in liquid culture is largely due to the integrated result of its adsorption rate, latent period, and burst size, which may be modulated by the physiological state of the host culture. Plaque formation is an analogous but not identical process. The spatial structure imposed by the soft agar overlay and its effects on phage diffusion play significant roles in the formation of plaques that can be observed by the unaided eye [26,45]. Phages with lower adsorption rates can actually produce more robust plaques, which is observed in the case of coliphage lambda PaPa and produces larger and more visible plaques due to the loss of its side tail fibers [46].

Strongly negative difference scores (i.e., less than ~−100) tended to be produced when a phage was more effective against a test strain than on its own host in the microtiter assay since all scores were normalized to the result on their propagation host. This result produces an over-unity microtiter assay score that can exceed even the highest possible score from the spot assay, which turns into a strongly negative difference score. In the case of most intermediate negative difference scores (i.e., from −20 to ~−100), the phage produced only a weak signal in the spot assay but was able to strongly control bacterial growth in the microplate assay. Hyman and Abedon [10] suggested that liquid culture-based host range methods can be used to determine the host ranges of phages with poor ability to form plaques on solid media. Phages with poor diffusion through soft agar overlays, very high adsorption rates, or long latent periods would be expected to produce smaller plaques with a potential impact on plaque-based measurements of phage sensitivity [26].

## 4. Conclusions

The majority of host range results produced by two methods agreed with one another and the microtiter host range assay was generally able to determine the phage host range at the same level of sensitivity as the conventional agar overlay spot method. This result indicated that the microtiter plate method developed here could serve as an alternative to the conventional agar overlay spot method for determining the phage host range. Compared to the traditional overlay method, however, the microtiter assay was able to provide more information on the phage-host interaction including the phage host range, virulence, and potentially the development of phage resistance in a high-throughput format. This type of assay represents a useful initial step in screening phage collections for therapeutic use and could guide phage choice in conjunction with additional information on the phage’s biology such as receptor use, cross-resistance patterns, and performance in animal models. In measuring two phage concentrations per phage-host pair, the method as described here can accommodate 32 assays per 96 well plate (two phage concentration plus positive control per combination). If a single phage concentration is tested, one plate can hold 48 assays. The host range results produced by the microtiter assay were interpreted and calculated automatically by the plate reader and computer, which reduces potential human errors generated by manual visualization of plaques in the spot assay. Compared to the MIC-like host range assay conducted by Vipra et al. [31], this method also prevents potential false negatives due to the development of phage resistance since it measures bacterial growth at short time intervals instead of at a single end-point [10].

In a study exploring using the concept of MIC to examine the host range and virulence of *Salmonella* phage, phage P16 was able to produce similar results against a panel of *Salmonella* strains in an agar overlay spotting method, but MICs of the phage against the same strains could vary by 10 million-fold in a liquid culture assay [31]. The tendency of spot assays to overestimate phage virulence was also observed by Henry et al. [15] in which phage PhiKZ displayed a greater-than-unity EOP of 1.2 but performed poorly in liquid culture. This phage also performed poorly in an in vivo model of phage therapy, which suggests that measures of phage virulence may be more useful than measures of plating efficiency when selecting phages for use in antibacterial applications. This concept is similar to the observations of Lindberg et al. [16] where phage fecundity in liquid culture was a strong predictor of phage in vivo efficacy in an insect model.

The evaluation of the phage host range in the liquid culture-based microtiter assay provides several advantages over the traditional spot assay. It is repeatable, eliminates the need for (often subjective) visual inspection of plaques, and provides information on both phage host range and phage virulence in a single assay. If desired, the large amounts of data generated by this method may be transformed into a single numerical value for inter-assay comparisons. Dosage effects were observed across the panel of phage-host combinations in the microtiter assay in which testing phages at higher titers resulted in an increased apparent bacterial sensitivity to phage. This observation demonstrates the importance of assay parameters on the observed phenotypes. Conducting such assays with at least two phage concentrations is beneficial for determining the phage host range and virulence characteristics.

## Figures and Tables

**Table 1:** Bacterial strains used in this study and their origins.

Strains	Serovars	Sources/References
USDA4	Anatum	T. Edrington (USDA)/[37]
FC1033C3	Anatum	Cattle Feedlot Environment/[37]
S2029C2	Anatum	Cattle Feedlot Environment/[37]
S2028C1	Anatum	Cattle Feedlot Environment/[37]
FD1001A1	Muenchen	Cattle Feedlot Environment/[37]
H2006-1	Cerro	Cattle Feedlot Environment/[38]
08-022	Dublin	S. Lawhon (Texas A&M Veterinary Medicine)
SGSC 2475	Enteritidis	*Salmonella* Genetic Stock Centre/(University of Calgary, CA)/[39]
3115	Enteritidis	T. M. Taylor (Texas A&M University)
SGSC 2480	Heidelberg	*Salmonella* Genetic Stock Centre (University of Calgary, CA)/[39]
3117	Heidelberg	K. Cummings (Texas A&M Veterinary Medicine)
USDA3	Montevideo	T. Edrington (USDA)/[37]
H1042-3	Montevideo	Cattle Feedlot Environment/[38]
USDA2	Newport	T. Edrington (USDA)/[37]
10-014	Newport	S. Lawhon (Texas A&M Veterinary Medicine)
330-1	Reading	S. Lawhon (Texas A&M Veterinary Medicine)
USDA1	Typhimurium	T. Edrington (USDA)/[37]
3116	Typhimurium	T. M. Taylor (Texas A&M University)
LT2	Typhimurium	American Type Culture Collection/ATCC 19585
USDA5	Kentucky	T. Edrington (USDA)

**Table 2:** Bacteriophages used in this study, their propagation hosts, and their origins.

Phages	Propagation Host	Source
Sasha	FC1033C3	Cattle Feedlot Environment/[37]
Season5	S2029C2	Cattle Feedlot Environment/[37]
Season6	LT2	Cattle Feedlot Environment/[37]
Sergei	FC1033C3	Cattle Feedlot Environment/[37]
Season12	USDA2	Cattle Feedlot Environment/[37]
Season13	LT2	Cattle Feedlot Environment/[37]
Munch	USDA3	Cattle Feedlot Environment/[37]
Minion	S2028C1	Cattle Feedlot Environment/[37]
Mecon	FD1001A1	Cattle Feedlot Environment/[37]
Season24	USDA4	Cattle Feedlot Environment/[37]
Morel	S2028C1	Cattle Feedlot Environment/[37]
Season27A	FC1033C3	Cattle Feedlot Environment/[37]
Sw2	FC1033C3	Municipal wastewater influent, TX
FelixO1	LT2	*Salmonella* Genetic Stock Centre (University of Calgary, CA)
Melville	USDA2	Municipal wastewater influent, TX
